# Urine-derived cells for human cell therapy

**DOI:** 10.1186/s13287-018-0932-z

**Published:** 2018-07-11

**Authors:** Nimshitha Pavathuparambil Abdul Manaph, Mohammed Al-Hawaas, Larisa Bobrovskaya, Patrick T. Coates, Xin-Fu Zhou

**Affiliations:** 1 Central Northern Adelaide Renal and Transplantation Service, Royal Adelaide Hospital, Adelaide, 5000 South Australia; 2 School of Pharmacy and Medical Sciences, Sansom Institute, University of South Australia, Adelaide, 5000 South Australia; 3 School of Medicine, Faculty of Health Sciences, University of Adelaide, Adelaide, 5000 South Australia

**Keywords:** Urine, Stem cells, Renal cells, Differentiation, Therapy

## Abstract

Desirable cells for human cell therapy would be ones that can be generated by simple isolation and culture techniques using a donor sample obtained by non-invasive methods. To date, the different donor-specific cells that can be isolated from blood, skin, and hair require invasive methods for sample isolation and incorporate complex and costly reagents to culture. These cells also take considerable time for their in-vitro isolation and expansion. Previous studies suggest that donor-derived cells, namely urine stem cells and renal cells, may be isolated from human urine samples using a cost-effective and simple method of isolation, incorporating not such complex reagents. Moreover, the isolated cells, particularly urine stem cells, are superior to conventional stem cell sources in terms of favourable gene profile and inherent multipotent potential. Transdifferentiation or differentiation of human urine-derived cells can generate desirable cells for regenerative therapy. In this review, we intended to discuss the characteristics and therapeutic applications of urine-derived cells for human cell therapy. Conclusively, with detailed study and optimisation, urine-derived cells have a prospective future to generate functional lineage-specific cells for patients from a clinical translation point of view.

## Background

Cell therapy aims to restore diseased or injured tissues by replacing lost cells with functional cells to re-establish normal function [1]. Generally, functional cells for therapy are generated from donor-derived somatic cells or stem cells by differentiation. The method of sample collection for the widely used donor-derived cells such as keratinocytes, adipose-derived stem cells, and mesenchymal stem cells (MSC) require needle insertion, biopsy, or physical dislodgement by scraping. Although these cells can be cultured and differentiated successfully with various protocols, sample isolation is quite complicated, and the cells also take considerable time for expansion. On the other hand, cells isolated from human urine samples, urine stem cells and renal cells, do not require such invasive methods for sample collection [2]. Urine cells can be isolated using a relatively simple method and be expanded easily. In addition, the possibility of generating cells from urine samples makes the human urine-derived cells an attractive alternative choice for cell therapy [3, 4]. However, few manuscripts have been published thus far to describe the use of these cells in cell therapy. In this review, we focus on some of the significant aspects of urine cells that can be utilised for different patient-specific regenerative therapies.

## Isolation of urine-derived cells

The ease of isolation is the main advantage of urine-derived cells compared with all other donor-related samples. Adipose-derived stem cells, hair cells, and mesenchymal stromal cells require liposuction or invasive methods for sample isolation. Amniotic and umbilical cord cells are neither easily accessible nor suitable for auto-transplantation. These hurdles make the cost of biopsy for the aforementioned cells high, ranging from $150 to 500 (per sample) and the procedure requires hospital admission, physician consultation, and surgical procedure. On the contrary, collection of urine samples does not require such specialised procedures. Urine-derived cells can be isolated at a cost less than $70 (per sample) by simple centrifugation of the samples to sediment the cells and by seeding them onto normal culture plates without special substrates [2]. Following isolation, they may be expanded with minimal labour to generate a considerable number of cells (an average of 2–7.2 cells/100 ml sample during initial isolation which can be expanded to 0.5–1 million by 10–15 days) that express a wide range of markers [3].

Two significant types of undifferentiated donor-derived cells can be generated from human urine. Firstly, urine stem cells (USC) are progenitor cells that can be converted into cells of multiple lineages [5]. USC supposedly originate from the kidney due to their high gene expression for kidney cortex markers [6]. Studies have demonstrated the presence of “Y chromosome” in a female patient who received a male kidney transplant, suggesting a cell origin from the kidney [6]. USC can be expanded to generate more than a million donor specific cells by two consecutive subcultures after their in-vitro isolation. The second type, so called “renal epithelial cells” or “renal cells” are considered less potent than USC in terms of gene expression and in-vitro expandability [4]. Compared with the renal cells, USC can generate more donor-specific multipotent cells by cell culture.

The practical method of urine cell isolation includes the centrifugation of the collected sample at 400 g followed by an antibacterial wash of the pellets (to avoid contamination) and later seeding them onto culture plates with medium. For USC, the sedimented cells are seeded and subcultured with 10% fetal bovine serum containing Dulbecco’s modified Eagle’s medium (DMEM), DMEM/nutrient mixture F-12 (DMEM/F-12) and keratinocyte serum-free medium with supplements. USC can be preserved inside the collected urine samples for 24 h without any considerable loss of viability [7]. USC have been isolated from patients with a number of disease conditions including haemophilia A, B-thalassemia, Duchenne muscular dystrophy, systemic lupus erythematosus, epidermolysis bullosa, bladder cancer, and neurological disorders (Parkinson’s disease, acute myeloid leukaemia) [8–12]. Renal cells have been isolated from healthy donors only, and thus less information is available on the isolation of renal cells from diseased patients. Unlike USC, renal cells are seeded and cultured using renal epithelial cell growth medium. After isolation and expansion and preservation using dimethyl sulphoxide, USC and renal cells can be stored in liquid nitrogen, holding a strong revival capability for future applications without any considerable loss of viability [8].

Podocytes and proximal tubule epithelial cells (PTEC) are the other two types of cells that can be isolated from human urine samples. However, podocytes and PTEC are mature, differentiated cells and have less expandability and lifespan compared with the undifferentiated cells (USC and renal cells) that can be isolated from urine samples. In addition, the cells require immortalisation to maintain them in vitro. Because of the terminal differentiation, podocytes and PTEC are non-significant in terms of generating lineage-specific cells in considerable quantities for therapy and therefore are not discussed in this review.

## Characteristics of urine-derived cells

### Urine stem cells (USC)

Urine stem cells (USC) express a variety of markers for pericyte, MSC, and pluripotent stem cell markers (Table 1). Some of the key pericyte markers expressed by USC are CD224, CD146, platelet-derived growth factor r beta (PDGF-rβ), and neural/glial antigen 2 (NG2). The critical MSC markers such as CD44, CD73, CD90, and CD105 are highly expressed by USC. Previous studies suggest that USC are urine-derived MSC due to the high expression of the above markers [13]. In addition, USC express the pluripotent stem cell markers POU5F1 or octamer-binding transcription factor 3/4 (Oct 3/4), VMyc avian myelocytomatosis viral oncogene homologue (c-Myc), stage-specific embryonic antigen 1/4 (SSEA-1/4), and Kruppel-like factor 4 (Klf-4) [5]. Since urine contains different cell types, after sample isolation the aforementioned pluripotent marker expressions can serve as typical markers for the confirmation of USC isolation from any donor sample. In addition, USC express the renal cortex markers sine oculis homeobox homologue 2 (SIX2), neural cell adhesion molecule (NCAM), epithelial cell adhesion molecule (ep-CAM), and frizzled class receptor (FZD), suggesting their origin from the kidney [14–16]. Therefore, urine-derived stem cells may also be termed kidney progenitor cells or kidney stem cells [17]. Urine stem cells also express a small proportion of endothelial, epithelial, smooth muscle, and interstitial markers (Table 1), and the significant highlight of these cells is their high in-vitro expandability.

**Table 1:** Markers expressed by different urine-derived cells

Markers	Urine stem cells	Renal cells	Podocytes	PTEC	Reference
ESC/iPSC	Oct 3/4, Sox-2, c-Myc, Klf-4, SSEA-4, Tra-1-60, Tra-1-81	Sox-2	–	–	[3, 6]
MSC	CD29, CD44, CD54, CD73, CD90, CD105, CD166, STRO-1	–	–	–	[13]
Pericyte	CD24, CD133. CD140b, CD146, CD224, PDGF-rβ, NG-2	–	–	–	[46]
Haematopoietic stem cell	CD34, CD45, MHC-I	–	–	–	[13]
Interstitial cells	c-Kit	–	–	–	[3]
Renal tubular	CK-7	CK-7, SLC2A1	–	–	[32]
Fibroblasts	Vimentin, α-tubulin	Actin, Vimentin	–	–	[32]
Smooth muscle	α- SMA, Desmin	–	–	–	[6]
Urothelial	CK-13, CK-19, Uroplakin	CD 13	–	–	[46]
Endothelial	vWF, CD31	–	–	–	[27, 47]
Kidney-specific	Pax 2, Pax 8, Six 2, FZD, ep-CAM	L1CAM, NR3C2	–	–	[32, 46]
Membrane/tight junction	Zo-1, Occludin (traces)	β- Catenin, E-cadherin, Claudin 1	–	Zo-1, E-cadherin, MRP4 Oct-2 P-gp, BCRP	[32, 86]
Pancreatic	–	Sox-17, PDX1	–		[55]
Hepatic	–	Sox-17, AFP	–		[55]
Others			Podocalyxin, synaptopodin, GLEPP1, podocin	Collagen I αI, Collagen IV αI, fibronectin I, laminin 5	[86, 87]

Urinary stem cells (USC) stand superior in terms of the markers expressed. The gene profile of renal cells has not been extensively studied compared with USC and, therefore, detailed analysis of the markers needs to be carried out

See the abbreviations list for definitions of the marker acronyms

*ESC* embryonic stem cells, *iPSC* induced pluripotent stem cells, *MSC* mesenchymal stem cells, *PTEC* proximal tubule epithelial cells

USC have high expandability compared with other widely used stem cells such as bone marrow stem cells, blood progenitor cells, keratinocyte progenitor cells, umbilical cord stem cells or adipose-derived stem cells [18–21]. Urine stem cells may reach nearly 70 population doublings and have an average doubling time of 21–24 h. On the other hand, the doubling time of the aforementioned non-urine-derived cells are greater than 24 h and their method of isolation and culture incur considerable time as it involves complicated methods of sample processing. USC isolation does not involve such complicated procedures for sample processing. Furthermore, with the addition of serum-containing medium, more USC were cultured from one sample. Interestingly, Schosserer et al. reported that the USC isolation efficiency of male donors is better than female donors [22]. An important matter that requires attention here is the significant variability of gene expression in the isolated USC. A recent study on USC has demonstrated significant intra-variability of reported markers on subculturing [23]. Regardless, the cells maintain their multipotent nature in vitro.

Similar to induced pluripotent stem cells (iPSC), embryonic stem cells (ESC), and MSC, USC are multipotent [12, 24]. USC have shown the capability to generate cells from the mesoderm, endoderm, and ectoderm. Furthermore, USC secrete 25 different angiogenic paracrine growth factors as detected by human angiogenesis array, which include the key angiogenic factors such as vascular endothelial growth factor (VEGF), fibroblast growth factor (FGF), insulin growth factor (IGF), hepatocyte growth factor (HGF), platelet-derived growth factor (PDGF), and matrix metalloproteinases (MMP) [24, 25]. These angiogenic and immunomodulatory growth factors may play an important role in the vascularisation of cells derived from USC which, if subsequently transplanted, might influence the immune system of the hosts. Supplementation of the endogenous VEGF production of USC with growth factor beads have improved angiogenesis and stress urinary incontinence (SUI) in rodents by increasing vascularisation and survival of the transplanted cells [24, 26]. In addition, USC have improved the in-vivo vascularisation and growth if delivered through hydrogels, collagen, alginate microbeads, or three-dimensional biofilms in mice [24, 26–30]. The stem cells have restored sphincter function after vaginal distension injury in rats [31]. Thus urine-derived stem cells have great potential to generate donor-specific autologous cells for tissue repair for multiple degenerative diseases (Table 2).

**Table 2:** Differentiation capability of urine-derived cells and their potential application

Type of urine cell	Differentiated to	Markers expressed before differentiation	Markers expressed after transdifferentiation/differentiation	In-vivo testing reported	Potential application	Reference
Urine stem cells	Endothelial	vWF, CD31	KDR, VE-cadherin, FLT-1, eNOS	Yes	Renal reconstruction, angiogenesis, SUI, erectile dysfunction	[36]
	Uroepithelial	Uroplakin Ia	Uroplakin-III, AE1/AE3 and CK7	Yes	Urological reconstruction	[3, 6]
	Smooth muscle	α-SMA	Desmin, Myosin, Smoothelin,	Yes	Bladder reconstruction, Genitourinary repair	[5, 14]
	Myogenic	Nil	MyoD, Myogenin, Myf5, Myosin	Yes	Heart repair, SUI	[88, 89]
	Beta–like cells	Nil	PDX1	Yes	Diabetic treatment	[62]
	Osteogenic	Nil	Osteocalcin, Runx2, ALP	Yes	Bone tissue engineering	[21]
	Neuronal	Sox-2	GFAP, Nestin, NF-200, S100	no	Neural tissue engineering	[85]
	Chondrogenic	Nil	Sox-9, Collagen II, Aggrecan	Yes	Cartilage replacement	[22, 90]
	iPSC	Klf-4, Sox-2, Oct 3/4, c-Myc	Nanog	Yes (teratoma)	Disease modelling/drug screening	[91]
Renal cells	Neural stem cells	Sox-2	Nestin, Pax6,	Yes	Neurodegenerative disorders	[33]
	Beta cells	Sox-17, PDX1	NKX6.1, Insulin, C-peptide	Yes	Diabetic therapy	[55]
	iPSC	Unknown	Sox-2, Oct3/4, Klf-4, Tra-1, SSEA-4	Yes (teratoma)	Disease modelling/drug screening	[32]

Urine stem cells have been shown to generate differentiated cells for kidney, genitourinary, cartilage, bone, and cardiac repair

Renal cells have been utilised to generate differentiated cells such as beta cell and liver cells

Pluripotent stem cells have been generated from both renal cells and urine stem cells

See the abbreviations list for definitions of the marker acronyms

*iPSC* induced pluripotent stem cells, *SUI* stress urinary incontinence

### Renal cells

**Fig. 1:**
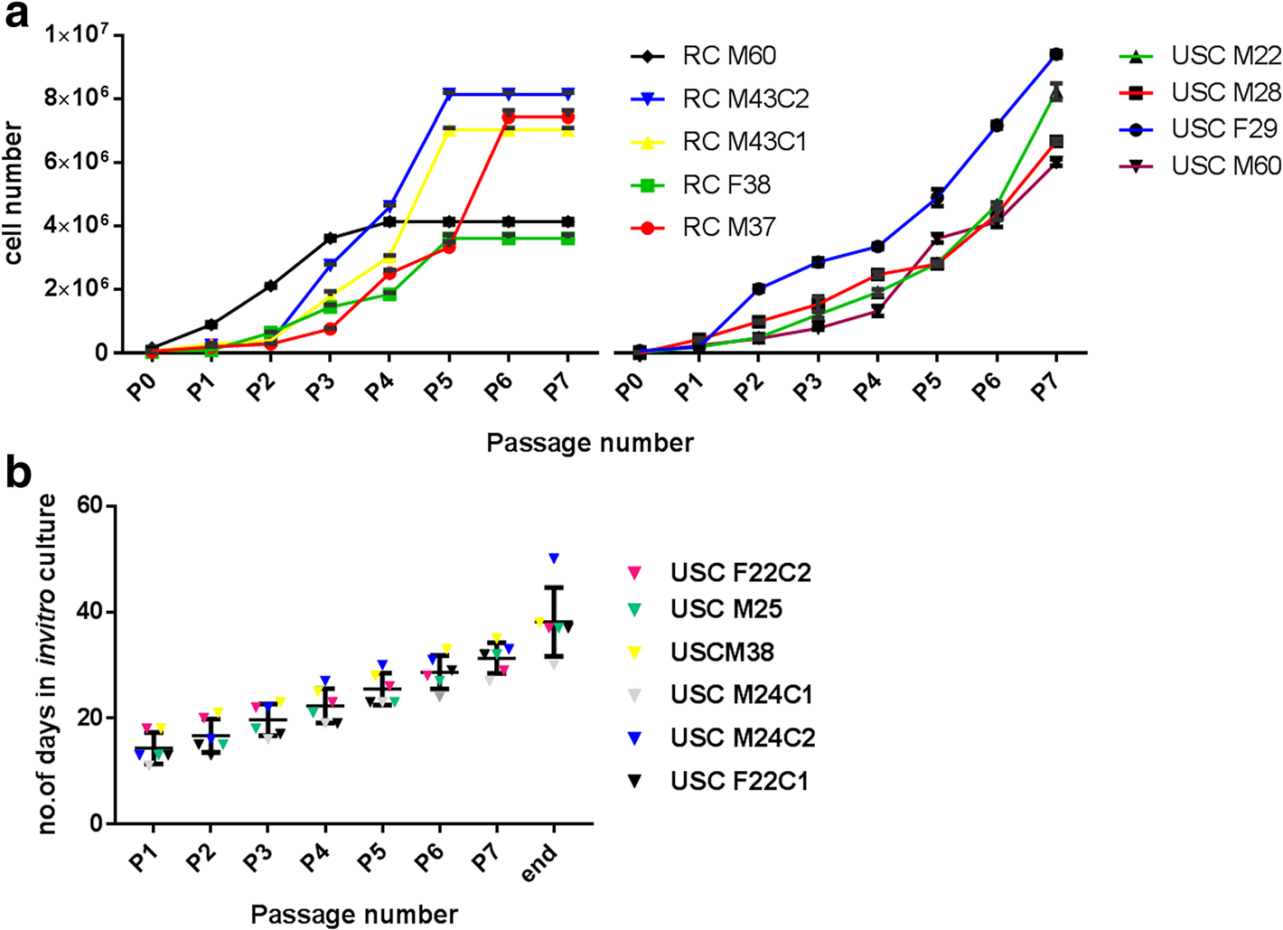
In-vitro characteristics of the urine-derived cells. **a** Growth curve analysis of renal cells (RC) and urine stem cells (USC) from different donors. Analysis reveals that USC have better expandability than renal cells. Renal cells demonstrated less expandability by passage 5. RC M60, M43, F38, and M37 indicate cells cultured from donors of the following ages (years)/gender: 60 (male), 43 (male), 38 (female), and 37 (male), respectively. C1 and C2 indicates the cell line number. USC M22, M28, F29, and M63 indicate urine stem cells cultured from donors of the following ages (years)/gender: 22 (male), 28 (male), 29 (female), and 60 (male), respectively. **b** Life-span of urine stem cells. Irrespective of the cells being from different donors, the isolated urine stem cells have been shown to generate viable cells up to 40–45 days

**Fig. 2:**
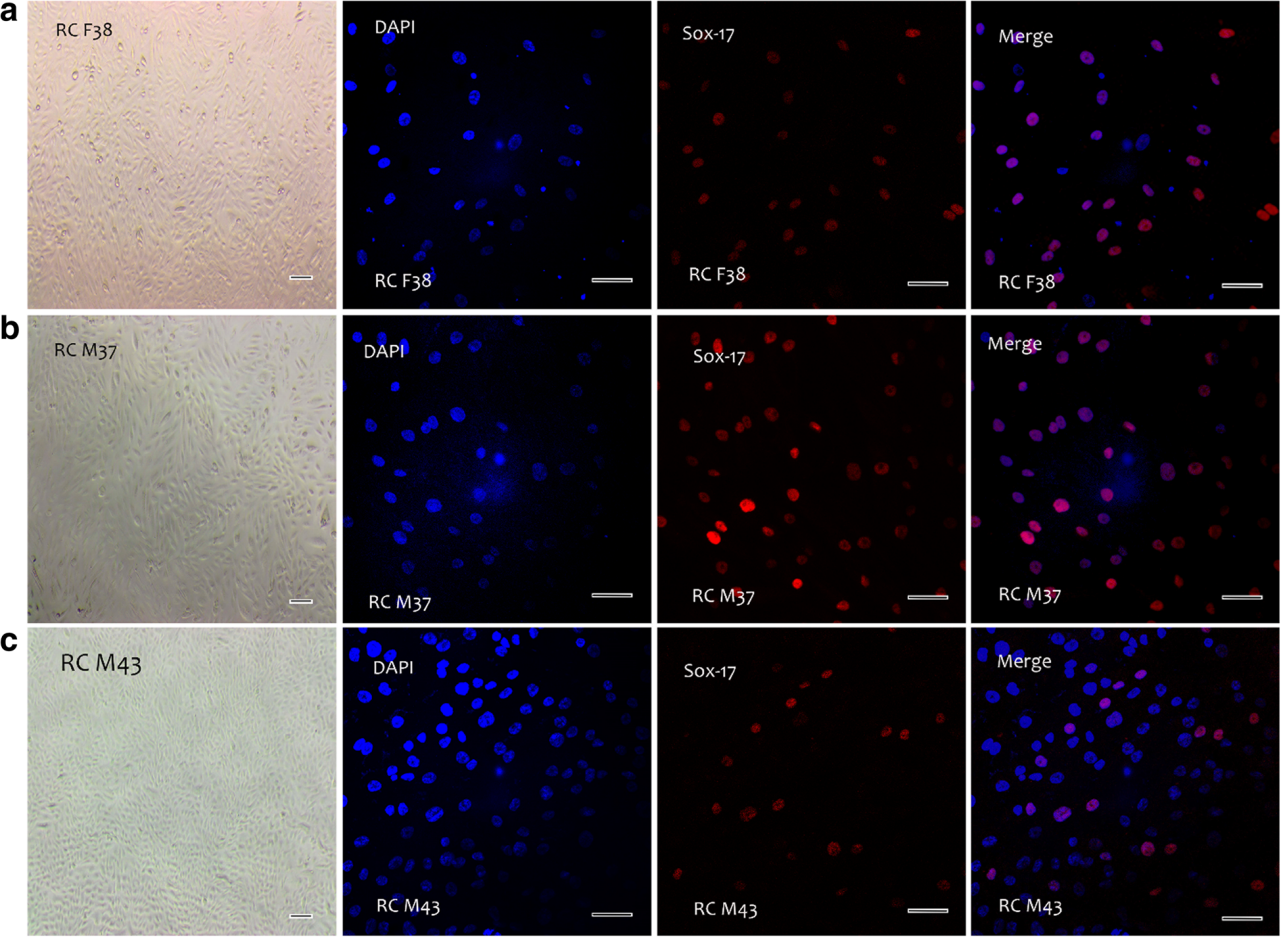
Sox-17 expression in renal cells. **a** RC F38, **b** RC M37, and **c** RC M43 indicate renal cells cultured from donors of the following ages (years)/genders: 38 (female), 37 (male), and 38 (male), respectively. The blue colour indicates nuclear staining of individual cells for all Sox-17 staining for respective donors. Depending on the quality of samples, the cells were positive for the endoderm marker Sox-17 (red colour) at varying levels. For the phase-contrast images, pictures were taken at 4× magnification and scale bar = 1000 μm. For fluorescent images, pictures were taken at 40× magnification and scale bar = 50 μm

Renal cells are considered as intermediate cells between kidney proximal tubular epithelial cells and fibroblasts (Table 1). Research indicates that renal cells express Beta-cadherin, E-cadherin, CD13, cytokeratin 7, zona occludens 1 (Zo-1), fibronectin, and vimentin [32]. They express some neuronal, beta cell, and hepatocyte markers (Table 1). The cell growth and in-vitro characteristics of renal cells are not known extensively in comparison with urine stem cells. However, from our in-vitro expansion studies of renal cells and USC, the isolated renal cells demonstrated less expandability than urine stem cells (Fig. 1). Nevertheless, irrespective of the donor sample and volume, urine stem cells demonstrated an in-vitro lifespan of approximately 40–45 days (Fig. 1). Renal cells derived from human urine samples were converted into neural stem cells by a non- integration-free method using small molecules [33]. The induced neural progenitor cells were converted into three different brain cell types (astrocytes, oligodendrocytes, and neurons), providing a safe and promising option for neurodegenerative diseases. In addition, the protocol does not incorporate any transcription factors and does not cause potential alterations in the genome. From our research, we have found out that the renal cells express the sex-determining region Y-related HMG box (Sox)-17 marker at high levels (Fig. 2), suggesting that they can be useful for generating endoderm-derived cells. Due to the high expression of the key endoderm marker Sox-17, renal cells can be a good source of donor-specific cells for liver, pancreas, or thyroid repair. However, extensive studies should be carried out on renal cells, as with USC, to understand their potential in terms of differentiation, gene expression, paracrine activity, and transplantation.

Above all, renal cells and USC can serve as the easiest cells to be isolated from any other well-studied donor cells and, consequently, urine-derived cells can serve as an ideal choice for generating differentiated cells for human therapy (Table 1).

## Therapeutic applications of urine-derived cells

Currently there is a lack of robust donor-derived cells that can serve as potent therapeutic cells for human therapy. Nevertheless, human therapy requires millions of cells ready for transplantation [34]. Hence, the choice of precursor cells is a significant challenge to achieve the cell numbers required for transplantation [18, 35]. In addition, the cells to be utilised for regenerative therapy have to meet other general stem cell characteristics. They should be harvested non-invasively from the donor and should be differentiated into multiple lineage cells in a reproducible manner. Moreover, the cells must be safe to perform an autologous transplant and should be generated according to Good Manufacturing Practise (GMP) guidelines. Urine cells also have other possible advantages from a therapy prospective. The ease of donor sample collection with minimal ethical issues makes them ideal for clinical applications. In addition, the low costs and the simple method of cell isolation is another advantage. Furthermore, there should be no immune rejection if used for regenerative transplants and it can be straightforwardly automated. Above all, the cells can be translated into commercial production and widespread clinical applications [3, 4, 6, 19, 20]. For the mass production of a wide range of differentiated cells (Table 2), urine-derived cells can serve as autologous cell therapy for erectile dysfunction, SUI, kidney bioengineering, cardiac and genitourinary repair, liver reconstruction, and neurodegenerative disorder treatment [2, 21, 36–38]. Currently, most of the investigated cells for the treatment of these diseases are generated from iPSC. However, extensive differentiation protocols combined with ethical issues makes iPSC more complicated for use in clinics.

### Renal reconstruction/kidney bioengineering

Generally, organ-specific cells are considered ideal for the treatment of the organ from which they are isolated or originate [39]. For this reason, urine stem cells will be the best option for genitourinary repair and kidney bioengineering. Urine cells have the potential to be differentiated into smooth muscle, myocytes, epithelial, and endothelial cells that forms most of the renal tissues. Three-dimensional arrangement of these cells can lead to the development of a kidney-like structure that can successfully function.

Presently, the concept of bioartificial kidneys are gaining popularity in terms of treating kidney failure. However, the cells used inside the device need to be effective in terms of removing the uremic toxins and to perform the complex glomerular functions [40]. Epithelial cells from the proximal tubule-derived cells are generally used for these devices [41]. USC and renal cells can serve as a good replacement for epithelial and proximal tubule cells in the bioartificial kidneys. In addition to epithelial and proximal tubule cells, endothelial and MSC have also been proven effective for reducing the progression of chronic kidney diseases in clinical trials [42, 43]. The transplanted cells have been shown to reduce inflammation, oxidative stress, and tubular injury via the release of cytokines and growth factors, thereby reducing the development and intensity of the disease [44, 45]. When combined with growth factors and delivered through biopolymers, urine stem cells have also shown to release similar cytokines (IGF and VEGF) and increase the vascularisation by angiogenesis, suggesting their promising future for treating kidney diseases [27]. As the kidney is a complex organ of 26 heterogeneous tissues, whether the differentiated urine cells can maintain functionality similar to the kidney requires extensive research.

### Genitourinary repair

Urine cells can be easily cultured and transplanted (with or without differentiation to endothelial cells) for genitourinary repair [46]. The endothelial cells expressed the key markers kinase insert domain receptor (KDR), vascular endothelial (VE)-cadherin, Fms-like tyrosine kinase 1 (FLT-1), and endothelial nitric oxide synthase (eNOS), suggesting functional properties of the differentiated urine cells. Research suggests that differentiated endothelial cells from urine stem cells demonstrate functional efficiency to treat end-stage bladder diseases or bladder pain syndrome [3, 47, 48]. Transfected urine cells with epithelium-derived factor as well as differentiated uroepithelial cells were proven to minimise erectile dysfunction and corrected the cavernous structure by nerve regeneration in rodents [49]. In addition, the skeletal progenitors differentiated from USC can be used to treat SUI patients for muscle regeneration [46]. The urothelial and skeletal differentiation induced the expression of uroplakin-III, the anion exchange proteins AE1 and AE3, cytokeratin (CK)7, and desmin, myosin, and smoothelin [6]. The significant challenge using urine cells in a reconstructive scenario will be if the cells are strong enough to withstand the urinary pressure and layered structure while maintaining functionality. Again, this is a combined effort between the cells and the biomaterial used for urinary conduits, and functional studies need to be carried out. However, biomaterials or scaffolds containing smooth muscle cells and urothelial cells differentiated from USC can be promising conduits for urinary reconstruction [46, 50].

### Cardiac repair

Heart failure mainly involves damage of the heart muscle leading to its improper functioning [51]. Currently, iPSC and ESC have been shown to generate cardiomyocytes by differentiation [52, 53]. Pluripotent cells are hard to obtain and take an extensive time for differentiation. Urine cells are easy to obtain and to differentiate in vitro [6]. Differentiated myocytes from urine cells have shown augmented expression of myofibrillar proteins and surface markers such as myogenic differentiation (MyoD) factor, myogenin, and myogenic factor 5 (Myf5) (Table 2) [6]. However, no further research has been carried out in terms of optimising the method to generate functional cardiomyocytes from USC. With proper differentiation and further screening of molecules, they can be further differentiated into considerable quantities of contractile and synchronised mature cardiomyocytes to repair the heart damage.

### Liver reconstruction

A large percentage of the human population has been affected by liver failure globally, and almost all cases require organ transplantation [54]. In cases of acute liver failure, transplantation is the only option. Due to donor tissue insufficiency or immunogenicity of the transplanted liver cells, most patients die. A possible alternative for transplantation is to provide patient-specific hepatocytes. Renal cells have already been transdifferentiated into insulin-secreting cells by small molecules [55]. Efficient transdifferentiation will be achieved if the parent cell used for transdifferentiation belongs to the same germ layer as the transdifferentiated cell [54]. As renal cells have high expression of the endoderm marker Sox-17 (Fig. 1), the transdifferentiation process to generate endoderm-derived cells (thyroid cells, liver cells, and pancreas) from the urine cells will be easy and efficient as both the renal cells (parental cells) and transdifferentiated (final) cells will share the same pedigree. Therefore, with proper screening of small molecules and further analysis, urine cells can be transdifferentiated into hepatocytes. Besides, the isolated urine cells behave like mesenchymal cells and MSC are highly flexible in generating hepatocytes in vitro [56–58].

### Diabetes treatment

Diabetes has become the fastest growing chronic condition, with the incidence increasing at a higher rate than other chronic conditions such as heart disease and cancer. By 2020, the World Health Organization (WHO) has envisioned that 380 million people will be affected with this disastrous disease [59]. Although islet transplantation has been carried out in patients using cells from cadavers, the quantity of cells required is not sufficient. They also require life-long immune suppression for graft survival in the receivers [60]. Current beta-cell transplantation requires a large number of cells (10,000 islet equivalent/kg of body weight) for successful replacement [61]. Using urine cells these numbers are achievable, and research also suggests that the renal cells can be efficiently differentiated into insulin-secreting cells by small molecules [55]. A single study involving urine cells has reported an efficient protocol to generate functional insulin-secreting cells from renal cells [55]. The cells express PDX1^+ve^/NKX6.1^+ve^/INS^+ve^ and exhibit glucose-stimulated insulin secretion. In another study, after transplantation undifferentiated urine stem cells were able to generate pancreatic and duodenal homeobox 1 (PDX1)-positive cells in vivo and to minimise the disease symptoms [62]. Surprisingly, the researchers used USC isolated from healthy individuals only for the study. A similar study needs to be performed using the urine cells isolated from urine samples of diabetic patients. This will also address the question whether there are considerable differences in the isolation efficiency, morphology, and gene expression of markers in cells isolated from diabetic patients, critical for developing the idea towards clinical application. Nevertheless, sample collection combined with minimal effort for culture, followed by efficient conversion into insulin-secreting beta cells for transplantation from urine cells can bring revolutionary changes in the field of diabetes treatment.

### Neuroregeneration

Clinical trials using stem cell-derived neurons for brain disorders are limited. Several studies have reported the generation of functional neurons from the in-vitro differentiation of pluripotent stem cells or neural stem cells [63–65]. However, pluripotent stem cell-based protocols take nearly 2 months to generate functional neuronal cells [66, 67]. In addition, after in vivo transplantation, the cells lose their functionality due to the damage in the complex dendritic structure after separating them from the in-vitro adherent culture. An alternative option is to generate immature cells capable of efficient maturation after transplantation. Urine stem cells treated with growth factors and cultured on laminin-treated plates have been reported to show efficient conversion into immature neuronal cells [68, 69]. Donor-specific immature cells grown in biomaterials such as hydrogels can be transplanted without much loss of structure and can further facilitate the integration and maturation of the cells in vivo. In addition, Cheng et al. formulated a small molecule cocktail to generate neural progenitor cells from renal cells which show functional properties in vitro (Sox2^+ve^/Pax6^+ve^ cells, alkaline phosphatase staining, electrophysiology recordings) and in vivo (transplantation and survival) [33]. This may well serve as a possible alternative to provide functional neurons in larger quantities without any considerable loss of cells during transplantation for neuroregenerative therapy. Again, the critical part of using the technology will be to generate specific types of neurons (for example, dopaminergic neurons for Parkinson’s disease) within the biomaterials. However, formulating a standardised protocol for USC differentiation within the biomaterial with a reliable outcome is challenging, but feasible.

### Bone engineering

Osteoblast grafts can be used as an effective solution for bone regeneration [70]. A human source of autologous cells in large numbers that can differentiate or can be differentiated into osteoblasts is critically important for engineering human bone grafts [70, 71]. However, extensive bone damage requires osteogenic cells that can be supplied in a three-dimensional biomaterial to support bone regeneration. Currently, ESC- and iPSC-derived cells are generally used for bone grafts. Donor-derived cells such as MSC and adipose-derived stem cells are also administered but, again, isolation of these sources is invasive and laborious. A single recent study involving a three-dimensional scaffold combined with urine cells has provided convincing evidence that the scaffold can repair critical bone defects in animals [21]. The cells can be maintained, delivered, and efficiently differentiated to provide replaceable bone cells in damaged areas for patients and therefore could be a viable option in future for orthopaedics [72]. Furthermore, urine stem cells can serve as a better option for bone transplants in children due to the efficient proliferative, self-renewal, and differentiation capability of USC to convert into osteoblasts.

### Muscle engineering

The “rice grain”-like urine stem cells were transdifferentiated into spindle-shaped skeletal cells on myogenic differentiation, with high levels of expression of skeletal muscle markers [6]. The differentiated cells continue to proliferate and generate further skeletal cells on subculture [73]. Extensive clinical trials have been carried out using stem cells for muscle cell therapy, and the administration of stem cells was proven to repair the damage to some extent. Permanent repair is possible, but may require more than one transplant using autologous, self-renewing functional cells [74]. In addition, very few studies have been conducted in the area of paediatric Duchenne Muscular Dystrophy. Transplantation of differentiated muscle cells from urine cells can be more effective than the current methods of transplantation (from iPSC or ESC differentiation) where the cells provided are terminally differentiated without further proliferation in vivo. Urine cells can not only survive but can also proliferate moderately and generate more differentiated cells than iPSC and ESC [6]. Furthermore, paediatric treatment can be more effective due to the naive nature of urine cells isolated from them [13]. Interestingly, a recent study reported that urine cell generation from new-born infants has a low efficiency of isolation [75]. In that case, standardised protocols for isolation and differentiation need to be established to obtain reliable outcomes.

### Paediatric treatment

The growth of urine cells is age-dependent on the donor sample. Studies have shown that cells isolated from young children (not infants) have better proliferative and differentiation capability [72]. Also, the level of expression of each marker varies during subculture of adult urine cells [23]. Due to the high expression of MSC markers, research suggests that urine stem cells are mesenchymal cells derived from urine [22]. Clearly, MSC stand out in terms of clinical trials for a number of diseases [76, 77]. MSC are well known to impart minimal immune reaction after transplantation, making them currently the ideal source of cells for therapy. Interestingly, when mixed with allogenic blood cells, USC induced a smaller population of CD80/CD86^+^ cells (surface receptor cells which activate T-cells creating an immune reaction) compared with MSC [78]. This indicates that USC have better immune tolerance and are a very good alternative to or better than MSC for regenerative therapy. Besides, the isolation of MSC is invasive and costly [79]. Urine stem cells can be a cheap and affordable alternative for mesenchymal cells for human therapy [13]. Also, USC have better expandability than MSC [7]. Paediatric and adult urine can be the ideal choice for generating a donor cell bank such as blood, umbilical cord, or skin biopsy for future application [13]. In fact, it will be better than any other adult sample in terms of generating ample cells for storing since studies have shown that the cells isolated from young children have better proliferative and differentiation capability [72]. Although urine-derived cells can serve as donor-derived MSC, extensive pre-clinical studies in terms of gene expression, differentiation, expansion, and immune reaction need to be carried out. Nevertheless, MSC are multipotent cells that can be subcultured from different sources such as blood, bone marrow, umbilical cord, skin, and fat [80]. Therefore, the possibility of a subpopulation within the isolated urine cells that exhibit the MSC characteristics is also unknown and needs further investigation.

### Disease modelling using urine-derived iPSC

iPSC have been widely used to study mechanisms of disease pathology and to find patient-specific cures by disease modelling [81]. Pluripotent stem cells have proven effective in finding the underlying causes for cardiovascular diseases (such as LEOPARD syndrome and hypertrophy) and neurodegenerative diseases (such as Alzheimer’s disease, schizophrenia, and amyotrophic lateral sclerosis) [81]. Drug screening using differentiated patient-specific iPSC provides better donor-specific efficacy than animal-based testing, and iPSC can be efficiently generated from urine samples using a volume as low as 30 ml [32]. Research suggests that the time taken to induce pluripotent stem cells from urine cells is shorter (2–3 weeks) than the time taken for blood cells, fibroblasts, or keratinocytes (3–4 weeks) [82]. This is related to the fact that the urine cells express the pluripotent markers at considerable levels, which may reduce the barrier to generate completely reprogrammed pluripotent cells. Furthermore, urinary iPSC were also derived from urine samples of patients suffering from rare genetic disorders such as fibrodysplasia ossificans progressive, paroxysmal kinesigenic dyskinesia, cryptorchism, Down syndrome, and type 2 long QT syndrome [75], suggesting its significant prospects in studying such rare diseases. Urinary iPSC show superior differentiation potential and, therefore, the cells can be used for disease modelling to understand the pathological problems related to the condition and drug screening studies for common as well as rare genetic diseases [75, 83]. Although urinary iPSC generation from urine samples of patients is feasible and attractive compared with other sources such as the blood, skin, and hair, some hurdles remain, such as retaining the epigenetic memory or mutation after reprogramming and performing quality testing (in terms of homogeneity of iPSC clones), as well as long-term subculture; these should be assessed before they proceed to the clinic. Also, current exclusion criteria of donor samples in clinical transplantology (chronically infected human immunodeficiency virus, hepatitis C virus, hepatitis B virus patient samples) can also make it harder to apply urinary iPSC generation universally for the common population.

## Conclusion

**Fig. 3:**
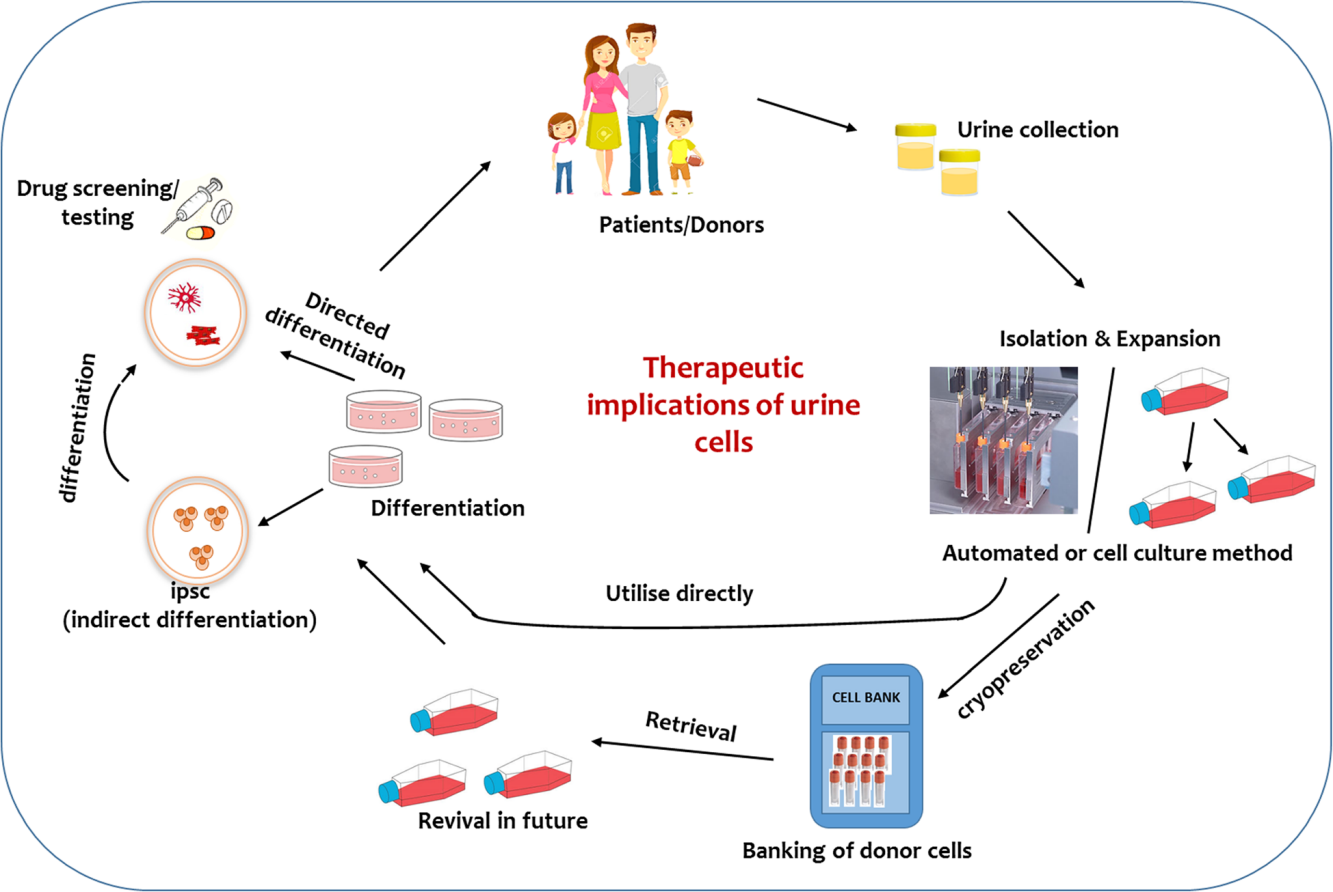
Future applications of urine cells in therapy. Donor-specific urine cells can be directly differentiated into organ-specific cells to transplant immediately or can be stored in cell banks for future revival, differentiation, and transplantation. ipsc induced pluripotent stem cells

Statistical analysis suggests that using the daily average volume of urine from an individual, 10 times the number of donor-specific urine cells required for an autologous tissue repair can be generated [4]. The automation of the urine cell cultures will make it easy to achieve the numbers for human transplantation. As a universal sample of collection, urine-derived cells can serve as an outstanding option for treatment of diseases such as those in the kidney, brain and paediatrics [84]. With more extensive study, urine cells can be employed as a preservation method for patient cells to serve as a cell bank for therapy and disease modelling study by generating iPSC (Fig. 3) [8, 85]. In addition, urine cells can also serve as a replacement for current MSC (derived through invasive methods such as bone marrow harvest and liposuction) to generate a donor bio-bank. However, this may require further expandability and qualitative studies, especially with the recent reports of considerable differences in isolation efficiencies and cell culture issues [23]. Although urine cells are an attractive solution, further testing of cells (especially renal cells) needs to be conducted to confirm the efficacy of the cells in terms of functional differentiation and long-term transplantation. The immune reaction of the differentiated cells after transplantation also need to be addressed. On critical analysis, we found significant inter-variability in the expression of reported markers and the reasons for such variations need to be addressed. One possible explanation for this heterogeneity in terms of gene expression can be due to the mixed nature of the isolated cells. This is also supportive of the fact that the human urine contains complex constituents and cells removed from the body. Sorting of the isolated urine cells at early passages can minimise the variation arising from the heterogeneous nature of urine cell cultures. Furthermore, for isolation and cell culture, we were unaware whether the urine storage conditions after collection (including adding serum to samples at room temperature and storing at 4 °C for 24 h to perform later isolation) and the duration of time the urine is held in the bladder before collection would affect the isolation. Upon extensive review, we have found that such conditions are important; for example, previous studies have reported variations in the quality of the cells (morphology and gene expression of USC markers) depending on different storage and sample collection conditions [7, 47]. An explanation is also unknown for the differences in the gene expression of the urine cell markers in different samples obtained from the same individual at different times (intra-variability). Furthermore, we also need to investigate if urine cell generation will be influenced by diet and medication. Generating clinically graded autologous cells from urine samples for cell therapy still needs comprehensive pre-clinical analyses to generate a standardised procedure. Conclusively, with detailed testing and optimisation, urine cells can serve as the future of regenerative therapy in terms of generating quantitative and qualitative cells for both organ reconstruction and to understand pathological mechanisms using disease modelling.

## Abbreviations

ACS: Adipocyte-specific cell surface protein
AE1: Anion exchange protein 1
AE3: Anion exchange protein 3
AFP: Alpha fetoprotein
ALP: Alkaline phosphatase
ALT: Alanine aminotransferase
α-SMA: Alpha smooth muscle actin
AST: Aspartate aminotransferase
BCRP: Breast cancer resistance protein
C/EPB α: CCAAT/enhancer-binding protein alpha
CK: Cytokeratin
c-Myc: VMyc avian myelocytomatosis viral oncogene homologue
eNOS: Endothelial nitric oxide synthase
ep-CAM: Epithelial cell adhesion molecule
ESC: Embryonic stem cells
FABP4: Fatty acid binding protein 4
FGF: Fibroblast growth factor
FLT-1: Fms-like tyrosine kinase 1
FZD: Frizzled class receptor
GFAP: Glial fibrillary acidic protein
GLEPP1: Designated glomerular epithelial protein 1
GMP: Good Manufacturing Practise
HGF: Hepatocyte growth factor
IGF: Insulin growth factor
iPSC: Induced pluripotent stem cells
KDR: Kinase insert domain receptor
Klf-4: Kruppel-like factor 4
L1CAM: L1 cell adhesion molecule
LpL: Lipoprotein lipase
MHC-1: Major histocompatibility complex class 1
MMP: Matrix metalloproteinases
MRP4: Multidrug resistance protein 4
MSC: Mesenchymal stem cells
Myf5: Myogenic factor 5
MyoD: Myogenic differentiation
NCAM: Neural cell adhesion molecule
NF-200: Neurofilament 200
NG2: Neural/glial antigen 2
NKX6.1: NK6 Homeobox 1
NR3C2: Nuclear receptor subfamily 3 group C member 2
Oct 2: Organic cation transporter 2
Oct 3/4: Octamer-binding transcription factor 3/4
Pax 2: Paired box 2
Pax 8: Paired box 8
Pax6: Paired Box 6
PDGF: Platelet-derived growth factor
PDGF-rβ: Platelet-derived growth factor r beta
PDX1: Pancreatic and duodenal homeobox 1
P-gp: P-glycoprotein
PPARϒ: Peroxisome proliferator-activated receptor
PTEC: Proximal tubule epithelial cells
Runx2: Runt-related transcription factor-2
S100: S100 calcium-binding protein
SIX2: Sine oculis homeobox homolog 2
SLC2A1: Solute carrier family 2, member 1
Sox: Sex-determining region Y HMG box
SSEA: Stage-specific embryonic antigen
STRO-1: S-adenosylmethionine synthase isoform type-1
SUI: Stress urinary incontinence
Tra-1: Tumour-related antigen 1
USC: Urine stem cells
VE: Vascular endothelial
VEGF: Vascular endothelial growth factor
vWF: Von Willebrand factor
Zo-1: Zona occludens 1
