# Correction to: Urine-derived cells for human cell therapy

**DOI:** 10.1186/s13287-018-0974-2

**Published:** 2018-08-22

**Authors:** Nimshitha Pavathuparambil Abdul Manaph, Mohammed Al-Hawwas, Larisa Bobrovskaya, Patrick T. Coates, Xin-Fu Zhou

**Affiliations:** 1 Central Northern Adelaide Renal and Transplantation Service, Royal Adelaide Hospital, Adelaide, 5000 South Australia; 2 School of Pharmacy and Medical Sciences, Sansom Institute, University of South Australia, Adelaide, 5000 South Australia; 3 School of Medicine, Faculty of Health Sciences, University of Adelaide, Adelaide, 5000 South Australia

## Correction

The original article [1] contained a small typo affecting the co-author, Mohammed Al-Hawwas’s name. This error has now been corrected.
